# Mild weather changes over China during 1971–2014: Climatology, trends, and interannual variability

**DOI:** 10.1038/s41598-019-38845-8

**Published:** 2019-02-20

**Authors:** Lijie Lin, Erjia Ge, Chongcheng Chen, Ming Luo

**Affiliations:** 1 School of Management, Guangdong University of Technology, Guangzhou, 510520 China; 2 Dalla Lana School of Public Health, University of Toronto, Toronto, ON Canada; 3 Key Laboratory of Spatial Data Mining & Information Sharing of Ministry of Education, National Engineering Research Centre of Geospatial Information Technology, Spatial Information Research Centre of Fujian Province, Fuzhou University, Fuzhou, 350116 Fujian China; 4 School of Geography and Planning, Sun Yat-Sen University, Guangzhou, 510275 China; 5 Institute of Environment, Energy and Sustainability, The Chinese University of Hong Kong, Sha Tin, NT, Hong Kong SAR, China

## Abstract

While previous studies largely focus on extreme events, little is known about the behaviors of mild weather, a positive and pleasant condition occurring frequently, directly associated with outdoor activities, and highly relatable to the public. Here we examine the climatological characteristics and long-term trends of mild weather over China during 1971–2014, as well as the possible linkage with the El Niño−Southern Oscillation. It is found that, on average, China experiences 94.5 days (25.4% of all days) of mild weather in a year, and the annual number of mild days increased by 1.02% per decade (3.73 days per decade) during 1971–2014, especially in summer (1.54% per decade), spring (1.49% per decade), and autumn (1.03% per decade). We also find that most parts of China have been experiencing increasing mild weather in 1971–1998 but decreasing in 1998–2014. Clustering analysis reveals six subregions that exhibit distinct mild weather behaviors. In particular, harsh seasons (i.e., summer of southern China and winter of northern China) are becoming even less pleasant. Besides these secular trends, it is also noticed that El Niño event in the preceding winter is followed by less pleasant spring and summer and more pleasant autumn and winter in most areas of China. The results reported here have significant implications for urban planners and governmental policymakers.

## Introduction

Mild weather describes a condition that is neither too cold nor too hot–a weather condition that could be considered as being “pleasant” or “comfortable”^1^. It occurs regularly in many parts of the world. During the past decades, extreme events such as heat waves, floods and droughts have increased in many regions, posing large impacts on public health, the multiple facets of the society, and the environment^2–8^. Nevertheless, little is known about the characteristics of mild weather. The focus on the mild weather is of great societal significance in a different way. The changes of mild weather are directly associated with human outdoor activities such as tourism, sports, building construction, and transport, and is highly related to the public^1^. For example, tourism and outdoor recreation all benefit from the consideration of the frequency and probability of mild and pleasant weather patterns in peoples’ plans. Understanding the changes in mild weather is valuable to a diverse range of businesses, industries, and agriculture^9^. However, previous studies on global or regional climate change mostly focus on extreme events, and how mild weather changes under global warming have not been understood clearly.

This paper conducts a systematic investigation of the changes of mild weather in China across space and time under global warming using daily observations at a network of 2,060 meteorological stations in mainland China from 1971 to 2014. In this study, the mean state of annual and seasonal mild weather frequency and mild days in different subregions of China are examined, the long-term trends of mild weather are investigated, and the possible association with the El Niño−Southern Oscillation (ENSO) is also examined. To the best of our knowledge, the current study is the first attempt to examine the spatio-temporal changes of mild weather in China.

## Climatology of Mild Weather Over China

**Figure 1:**
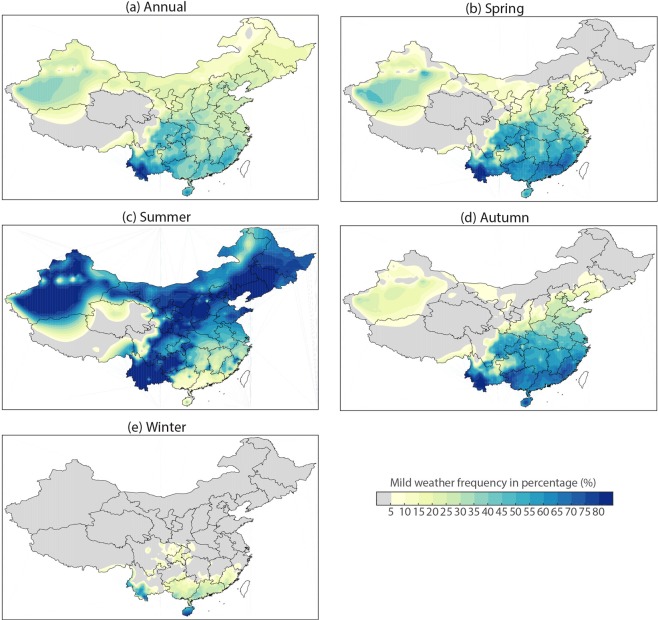
Multiyear climatological mean of (**a**) annual and (**b**–**e**) seasonal mild weather frequency in percentage in China over the period of 1971–2014.

The mean state (i.e., multi-year climatological mean) of mild weather in China is first examined. The spatial distribution of the climatological mean of annual mild weather frequency in percentage over the period of 1971–2014 is shown in Fig. 1. On average, China experiences an annual frequency of 94.5 mild days (25.9%) in a year (see also Table 1). The most frequent mild weather appears in southwestern regions, particularly in Yunnan Province (Fig. 1a). The hardest-hit regions are expected to be Tibetan areas, which also have the smallest population density and the highest elevation. Figure 1a also shows that southern, northwestern (e.g., southwest of Xinjiang province) and northern regions gain frequent mild weather days. However, the Yangtze River Basin and northeastern regions get relatively rarer mild weather, due to a humid and hot temperature in summer and very cold temperature throughout the year in these areas, respectively.

**Table 1:** Climatological mean mild weather frequency in percentage (%) in different subregions of China. SWC: Southwest China; TP: Tibetan Plateau; NC: Northern China; NCP: North China Plain; YRB: Yangtze River Basin; SC: South China.

	China	SWC	TP	NC	NCP	YRB	SC
Annual	25.9	61.2	4.0	25.9	37.8	38.0	45.2
Spring	23.1	66.6	1.0	12.6	36.1	46.8	61.1
Summer	54.2	87.9	13.9	80.5	78.0	45.9	21.3
Autumn	21.8	67.8	0.9	10.1	35.5	53.9	67.6
Winter	4.4	22.6	0.3	0.5	1.7	5.2	30.8

Mild weather in China exhibits strong seasonality (see Fig. 1 and Table 1). At the national scale, summer has more frequent mild weather; whereas, winter bears the most seldom. Also, the seasonality of mild weather varies in different subregions (Fig. 1). In spring, subregions including southern China and parts of northwest have more frequent mild weather. In summer, southern and southeastern regions, and Tibet experiences less frequent mild days than northern regions and parts of the southwest, due to hot summer climate in the south and cool in the north. Such pattern differs in autumn, with more frequent mild weather in the south and less in the north. In the winter season, most parts of China suffer from cold and windy conditions and gain much rare mild weather, with only small part of the south getting the slightly mild condition (mostly in Yunnan and Hainan provinces).

**Figure 2:**
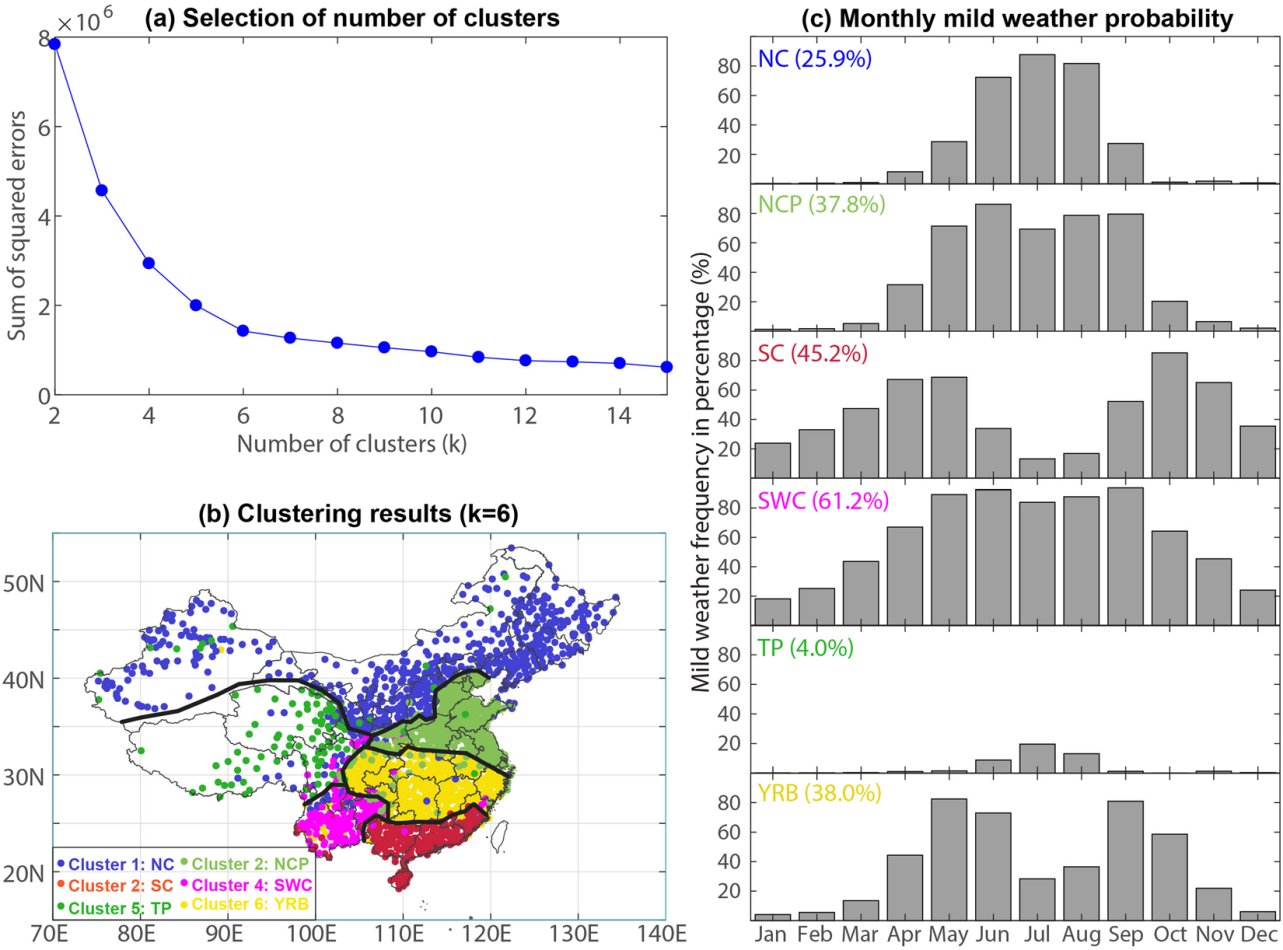
Clustering results of mild weather in China. (**a**) Sum of squared errors of *k*-means clustering of mild weather frequency in percentage at meteorological stations in China over the period 1971–2014. (**b**) Clustering results with k = 6. (**c**) Monthly mean mild weather frequency in different subregions with a colored number in percentage denoting the annual mean.

To examine the seasonal variation of mild weather in different regions of China, *k*-mean clustering^10^ is used to categorize the whole China into different subregions with distinct mild weather variations (Fig. 2). Six categories are identified (Fig. 2a,b), namely, Northern China (NC), the North China Plain (NCP), South China (SC), Southwest China (SWC), the Yangtze River Basin (YRB), and the Tibetan Plateau (TP). These categories well describe the regionalization map of mild weather in China. The seasonalities of the mild weather characteristics in these subregions are displayed in Fig. 2c.

These subregions exhibit distinct annual and seasonal variations in term of mild weather frequency (see Fig. 2c). Comparatively, SWC (particularly Yunnan province) has the highest proportion of mild weather, 61.2%, i.e., 223.4 days in a year, and TP has the lowest mild weather proportion (i.e., 4.0%) and the least mild weather days (i.e., 14.7 days in a year). People in NC and NCP experience 25.9% (94.6 days) and 37.8% (138 days) mild weather in a year, respectively. These subregions are also known as dry and cold areas. YRB has an average frequency of 138.5 mild days in a year, accounting for 38.0% of all days in a year, while SC has 164.9 days of mild weather in a year (i.e., 45.2%).

As shown in Fig. 2c, spring and autumn have the largest likelihood of mild weather in southern China (e.g., SC and YRB) while the northern regions (including NC and NCP) experience the most frequent mild weather in summer. On average, NC and NCP respectively gain 74.1 and 74.8 mild days in summer but only 0.4 and 1.6 mild days in winter (see Table 1), suggesting that winter in northern regions is uncomfortable for human beings. In summer, there is seldom mild weather in SC and YRB, where extremely high temperatures and heat waves frequently occur^7,10–12^. Due to the high altitude and colder temperatures, most frequent mild weather in TP appears in the summer season.

## Long-term Trends of Mild Weather Over China

**Figure 3:**
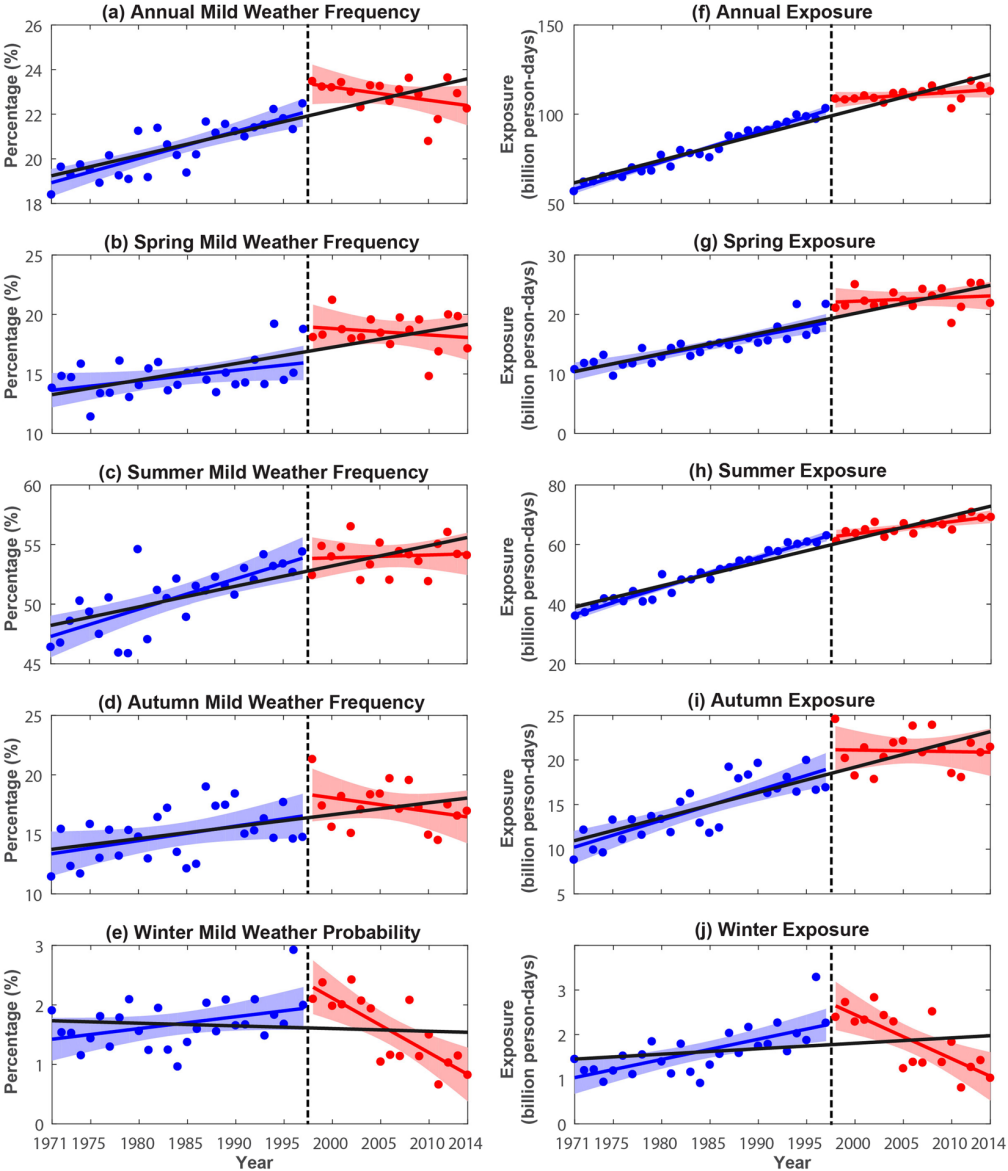
Yearly time series of the nationwide mean of (left) mild weather frequency in percentage and (right) population exposure to mild days in different seasons of China from 1971 to 2014. The black straight line indicates the corresponding trend in 1971–2014, blue line denotes the trend in 1971–1997, and red line denotes the trend in 1998–2014. Shading represents the prediction interval at the 95% level.

The nationwide areal mean of annual and seasonal frequencies of mild weather in China are depicted in Fig. 3. The yearly national population exposures to mild weather (in billion person-days) are also calculated (see Methods). A general increasing trend in both yearly mild weather frequency and population exposure during 1971–2014 can be observed (Fig. 3a,f). Yearly mild weather frequency (proportion) increases by 3.73 days (1.02%) decade^−1^ and yearly population exposure to mild weather increases by 14.1 billion person-days decade^−1^.

Though interannual variations for both annual and four seasonal means exist, mild weather in spring, summer, and autumn has significant increasing trend (i.e., *p*-value < 0.05) and winter exhibits a much slighter increase over the 1971–2014 period. More specifically, the mild weather in the summer season gets the largest increase of 1.54% (1.41 days) decade^−1^, and spring and autumn seasons have a relatively weaker increasing trend of 1.49% (1.37 days) and 1.03% (0.94 days) decade^−1^, respectively. Population exposures in summer, spring, and autumn also significantly increase over the whole period, i.e., 7.86, 3.37, and 2.84 billion person-days decade^−1^, respectively. Unlike the other three seasons, mild weather in the winter season has a weaker increasing trend of 0.03% decade^−1^, and the number of mild summer days increases by 0.03 days decade^−1^.

**Figure 4:**
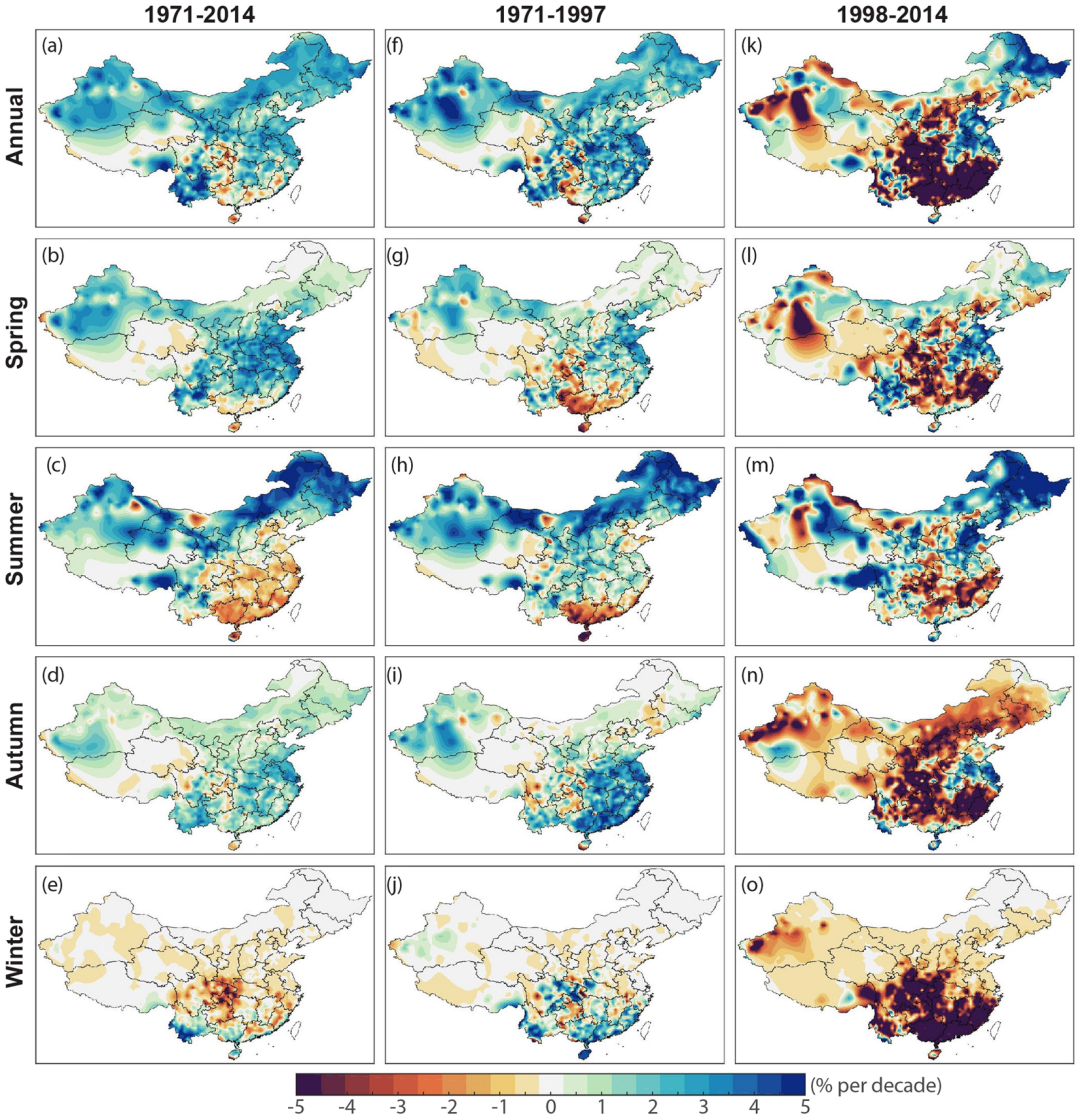
Spatial distribution of the long-term trends in annual and seasonal mild weather frequency in percentage over China during (left) 1971–2014, (middle) 1971–1997, and (right) 1998–2014. The trends for annual mean are scaled by 2.

The spatial pattern of the long-term trend of the mild weather frequency over the whole period of 1971–2014 are shown in Fig. 4a–e. Most regions except parts of central and southern China are getting more frequent mild weather in spring and autumn. Summer mild weather in southeastern China bears the prominent decreasing trend. Notably, the decreasing trend in summer SC and YRB are 1.63% and 0.82% decade^−1^, and these regions exhibit even less frequent mild weather in summer. In the winter season, many parts of China (except for parts of SWC and SC) are obtaining less mild weather.

**Figure 5:**
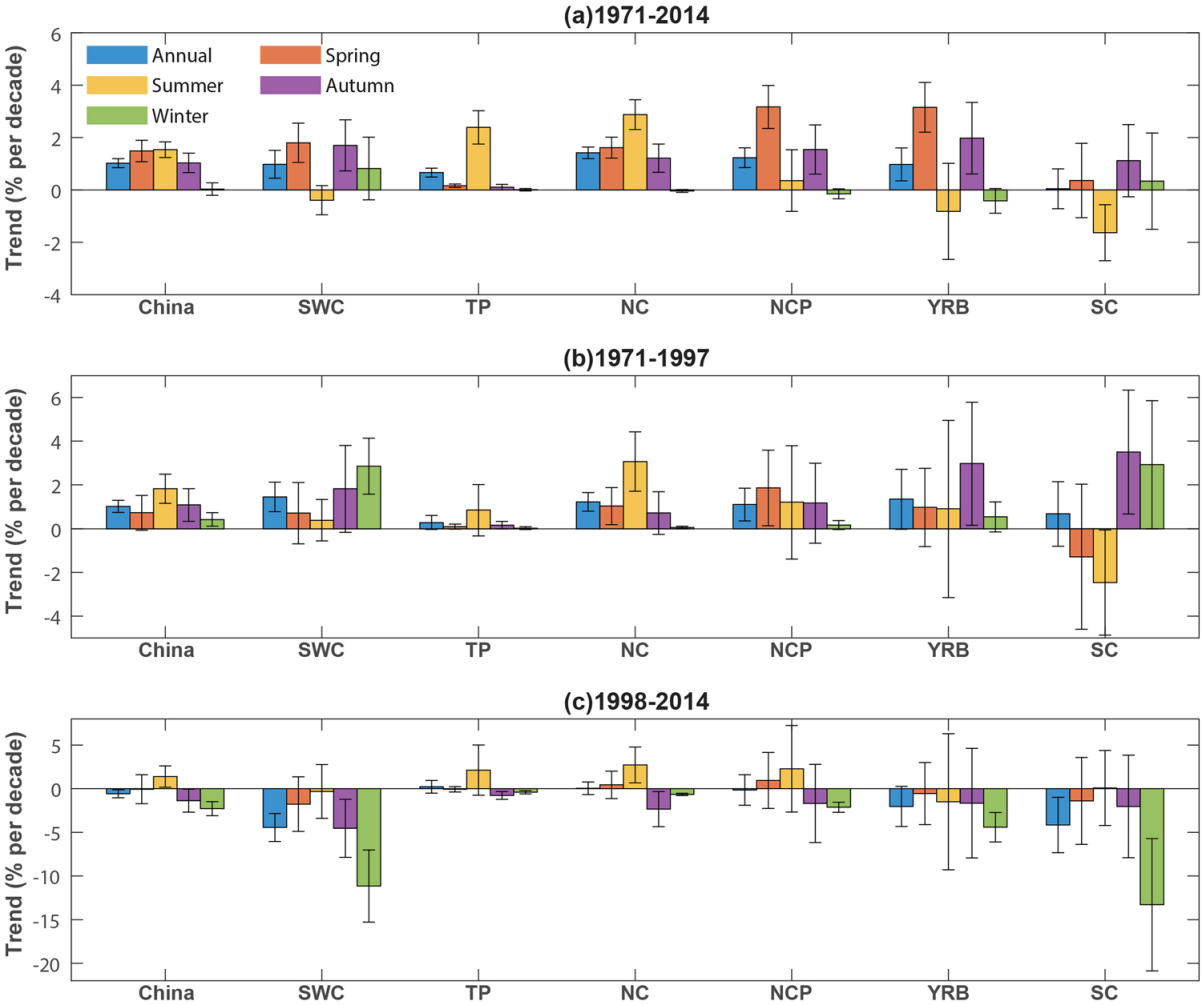
Charts showing the trends of mild weather frequency in different subregions of China in 1971–2014 (top), 1971–1997 (middle), and 1998–2014 (bottom). Error bar denotes the 95% confidence interval of the trend, that is, the interval containing the middle 95% of the slopes of lines determined by pairs of points in Sen’s slope estimation^15,16^.

It is also shown in Fig. 3 that, annually and for all four seasons, there is a noticeable increase during 1971–1997 but decrease during 1998–2014. This decreasing tendency corresponds to the global warming hiatus period^13–15^ and implies the response of China’s mild weather to global climate change. Besides the national mean, most time series for six subregions also show increasing trend before 1998 and decrease afterward (see Supplementary Information Figs S1–5 and Tables S2–3). Figure 4 depicts the spatial distribution of the trend magnitudes annually and for seasonal mild weathers in different subregions of China during 1971–2014, 1971–1997, and 1998–2014. The trends and significances in different subregions are also summarized in Fig. 5 and Tables S2–S3. During the subperiod of 1971–1997 (see Figs 4f–j and 5b), increasing trends in mild weather are dominant and are observed in nearly all seasons of all parts of China except for the spring of SC and summer of SC, SWC, and YRB. In these exceptional subregions, mild weather was becoming less frequent during 1971–1997. Over the most recent subperiod of 1998–2014, there are strong decreasing trends in most parts of China except for small parts of northern China. The decreasing tendencies are particularly strong in southern areas such as SC, SWC, and YRB.

## Contribution of Individual Variables to the Mild Weather Trend

**Figure 6:**
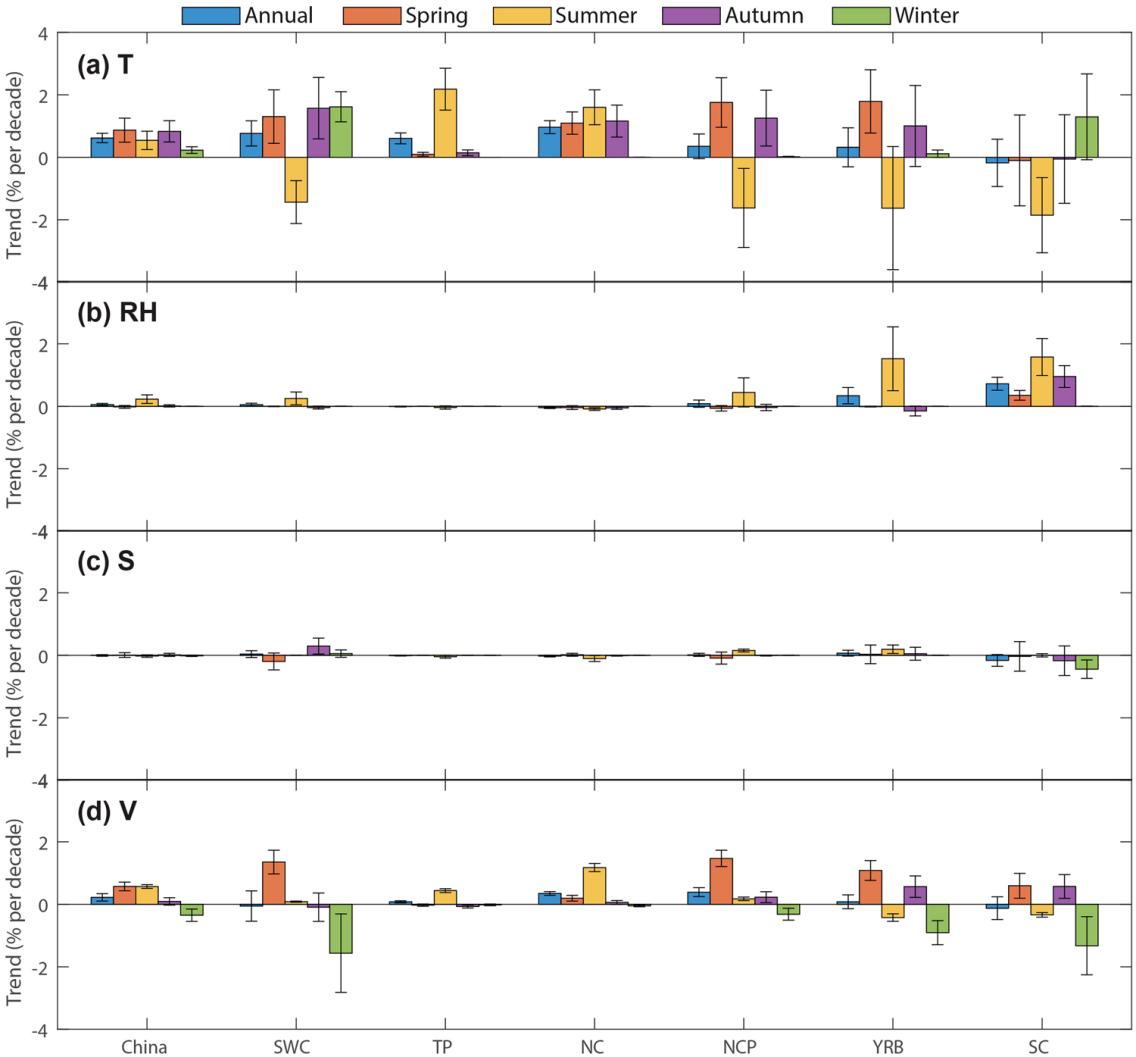
Effects of temperature (T), relative humidity (RH), wind speed (V), and sunshine duration (S) on the mild weather trends in different subregions of China. Error bar denotes the 95% confidence interval of the trend, that is, the interval containing the middle 95% of the slopes of lines determined by pairs of points in Sen’s slope estimation^15,16^.

To evaluate the relative contribution of individual variables including temperature (T), relative humidity (RH), wind speed (V), and sunshine duration (S) at the national and regional levels, we decompose the long-term trends in mild weather using a method similar to the ‘factor separation method’^16,17^ (see Methods). From Fig. 6, it is found that, at national and regional levels, T is the dominant factor contributing to the change of mild weather in China, followed by V and RH. T contributes to 0.7% (2.61 days) decade^−1^ increase in the annual mild weather proportion (days), and the effects of V and RH are 0.13% (0.46 days) and 0.06% (0.22 days) decade^−1^, respectively. T poses positive effects on mild weather in most regions but exerts essentially negative impacts in NCP and southern China (i.e., SC, SWC, and YRB, see Fig. 6a). Humidity has a substantial impact on mild weather in summer by intensifying summer heat stress^18^, particularly in NCP and southern areas (Fig. 6b) where the majority of China’s population lives. RH contributes to 0.89% (0.82 days), 0.67% (0.62 days), 0.50% (0.46 days) decade^−1^ of the decrease of mild weather in NCP, SC, and YRB, respectively. Surface wind speed also can reduce mild weather days in winter of these areas (Fig. 6c), since wind speed shows an overall weakening trend, which can prohibit the occurrences of winter wind chill. Compared with T, V, and RH, S (i.e., sunshine duration) has much smaller influences on mild weather change, mainly in reducing the mild weather in SC (Fig. 6d).

## Interannual Variability of Mild Weather and Linkage with ENSO

**Figure 7:**
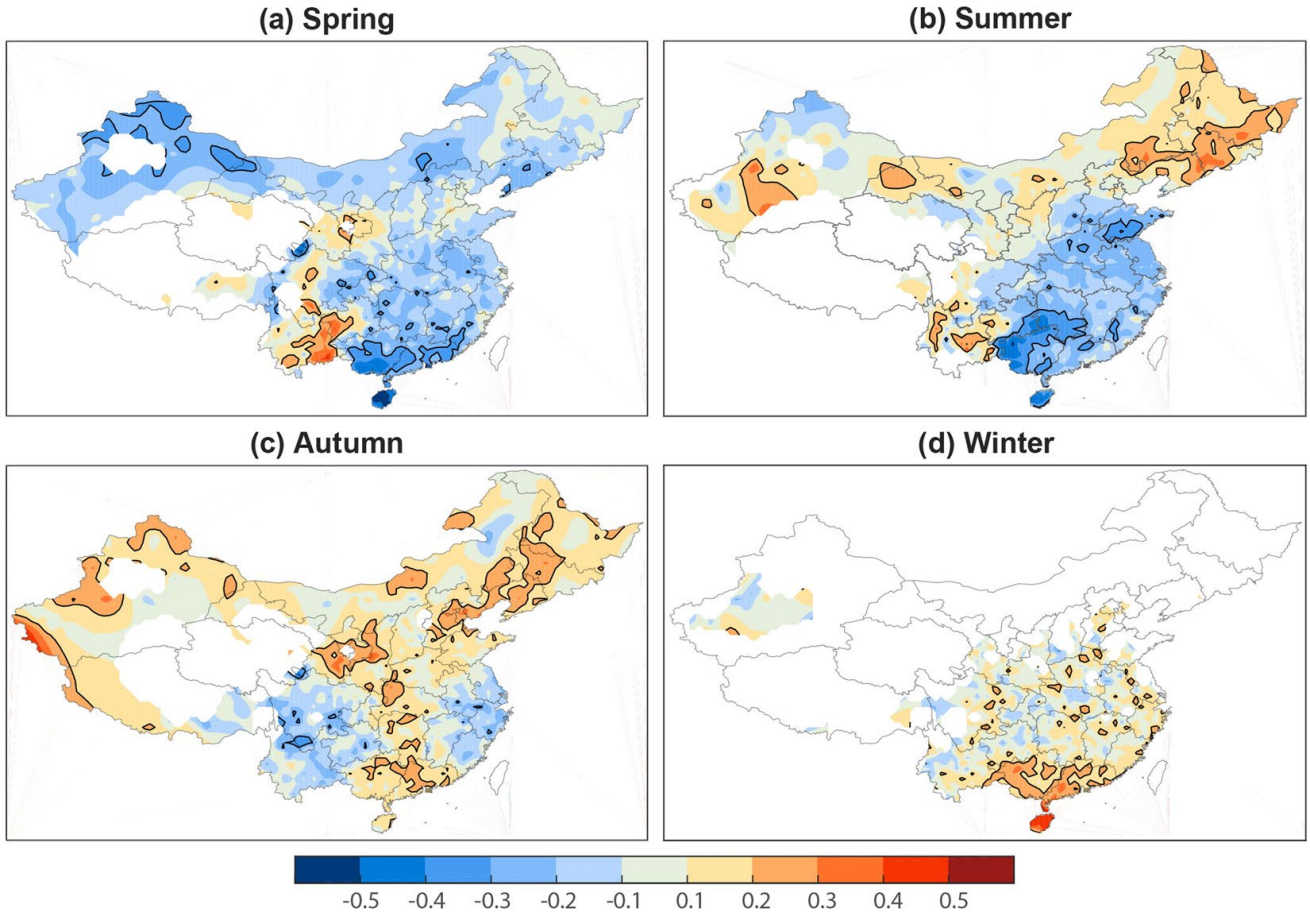
Spatial distribution of the correlation of mild weather frequency in percentage in different seasons with the preceding winter Niño3.4 index. Thick contour indicates the area with significant correlation at the 0.05 level.

In addition to the long-term trend in mild weather, its interannual variabilities and possible linkage with ENSO are now examined. Characterized by anomalous sea surface temperature in the tropical Pacific Ocean, ENSO is known as an important climate mode at the interannual scale. ENSO normally reaches its mature phase in the winter and has significant effects on the following summer high-temperature extremes in China^19–22^. It is likely that ENSO may also have a close association with mild weather conditions at interannual scale^23^. To examine the possible linkage between the preceding ENSO events and mild weathers, we calculate the correlation between the mild weather frequency in four seasons of China and the preceding winter Niño3.4 index. These results are shown in Fig. 7.

As Fig. 7 shows, the preceding ENSO events display close associations with mild weather in China, and these associations vary in different parts of China and differ from season to season. In particular, positive ENSO phases (i.e., El Niño) are followed by reduced mild weather in the following spring in most parts of China except for SWC while negative ENSO episodes (i.e., La Niña) may increase mild weather (Fig. 7a). These patterns are especially prominent in southern areas. For mild weather in the summer season (Fig. 7b), winter ENSO is accompanied by increasing mild weather in western and northern parts and decreasing in southeastern areas. As suggested by Luo and Lau^21^, mature El Niño event in the preceding winter induces a westward-displaced western North Pacific (WNP) subtropical high and an enhanced WNP anticyclone, thus increasing the occurrences of heat waves in southern China; whereas La Niña events weaken the heat wave activity there. As a result, mild weather in this area is decreased (increased) during El Niño (La Niña) decaying summer. As shown in Fig. 7c,d, the mild weather frequency in most parts of China exhibits positive correlation with the Niño3.4 index, suggesting that El Niño (La Niña) events are followed by increased (decreased) mild weather. Previous studies reported that the East Asian winter monsoon circulation tends to be weaker during El Niño and stronger during La Niña winter^24–26^. Therefore, a weakened (strengthened) winter monsoon exhibits warmer (cooler) surface temperature, thereby increasing (decreasing) the frequency of mild weather in late autumn and winter seasons.

## Summary and Discussion

In this study, we probe into China’s climate change from a different perspective by focusing on the mild weather, in terms of its climatology, secular trend, and possible linkage with ENSO. By examining the changes of mild weather conditions at more than 2,000 stations across the China over the period 1971–2014, it is found that China experiences an average frequency of 94.5 mild days (accounting for 25.9% of all days) in a year and summer season has the most frequent mild weather while winter bears the fewest. Based on the seasonalities of mild weather at different locations, we identify six subregions that exhibit distinct mild weather characteristics.

It is observed that the yearly frequency of mild days increases by 3.73 days decade^−1^ and the yearly percentage of mild weather increases by 1.02% decade^−1^ during 1971–2014. These trends are especially stronger in summer, spring, and autumn seasons. Spatially, the increasing trends are more prominent in northern than southern areas, suggesting that these people in northern China are getting more increasingly frequent mild weather. Nearly all parts of China have increasing mild weather in spring and autumn. More importantly, southern areas are experiencing reduced mild weather in the summer season, and northern regions tend to have decreasing trend in winter (Table 2). It indicates that harsh seasons (i.e., summer of the south and winter of the north) are becoming even less pleasant. In addition to these long-term trends, we also notice that El Niño winter is likely followed by less pleasant spring and summer and more pleasant autumn and winter.

**Table 2:** Trends of mild weather frequency in percentage (unit: % per decade) in different subregions of China during 1971–2014.

	China	SWC	TP	NC	NCP	YRB	SC
Annual	**+1**.**02**	**+0**.**98**	**+0**.**66**	**+1**.**42**	**+1**.**23**	**+0**.**97**	**+**0.04
Spring	**+1**.**49**	**+1**.**80**	**+0**.**16**	**+1**.**61**	**+3**.**17**	**+3**.**15**	**+**0.36
Summer	**+1**.**54**	−0.39	**+2**.**39**	**+2**.**88**	**+**0.36	−0.82	**−1**.**63**
Autumn	**+1**.**03**	**+1**.**70**	**+**0.10	**+1**.**21**	**+1**.**54**	**+1**.**98**	**+**1.12
Winter	**+**0.03	**+**0.82	**+**0.01	−0.04	−0.15	−0.42	+0.33

Bold indicates significance at the 0.05 level.

Note that the definition of mild weather is based on temperature, relative humidity, wind speed, and sunshine duration, as suggested by the China Meteorological Administration^27^. Besides these three elements, other variables such as precipitation (including both its timing and intensity) also play an important role in determining mild or severe weather^1^. Moreover, air pollution poses increasingly threat to the environment and living conditions. Heavy air pollution may also interact with synoptic meteorology and affect the mild weather frequency^28,29^. It is of great significance and interest to take these factors into account when defining mild or pleasant weather as perceived by human.

Base on clustering analysis (Fig. 2), we compare the mild weather changes in six different subregions that have distinct mild weather features. This regionalization is somehow general. Future studies should take more local characteristics into consideration. In this study, for example, we treat northwestern and northeastern China as a whole and notice that they show similar mild weather frequency. However, there are still differences in their climates, e.g., northeastern China has a wetter summer while the northwest is drier. In addition, due to the effect of local land-sea breeze, coastal areas are often more pleasant than inland areas. We do not analyze these differences in the current study since such analysis requires an even finer dataset that depicts detailed local features.

Also, it is of great interest to examine the possible local customs responding to mild and severe weathers. For instance, it is easier for the people in southern China familiar with humid hot weather to get accustomed to even less pleasant weather in summer than those in the north. Whether a unified mild weather definition suitable for all parts of China also need to be further evaluated. Moreover, given the increasing mild weather in winter (summer) and decreasing in summer (winter) in southern (northern) China, it is encouraged to consider the increase of the cooling (warming) surface during building constructions to mitigate these mild weather changes. It is also of significance to extend the existing studies on the physiological effects of climate and weather to consider the effect of mild and pleasant weather.

## Methods

### Data

Daily precipitation, temperature, relative humidity, wind velocity and sunshine duration in a homogenized dataset consisting of 2,474 stations in China are collected from China Meteorological Data Service Center (http://data.cma.cn) over the period of 1971–2014. A station is treated as missing if more than five days is missing in any five months, and a total of 2,060 stations are retained for analyses. Different seasons are defined as follows, spring: March–May, summer: June–August, autumn: September–November, and winter: December–February. The Niño3.4 index is obtained from the NOAA Climate Prediction Center at http://www.cpc.noaa.gov/products/analysis_monitoring/ensostuff/ensoyears.shtml.

### Definition of mild weather

The mild weather is defined by an official definition as suggested by the China Meteorological Administration^27^. A day is considered as mild if the daily temperature-humidity index (*THI*) is between 17 and 25.4 for May–October or the wind chill index (*WCI*) is between −299 and −100 for November–April (see Supplementory Table S1). Here, *THI* is an indicator of apparent temperature as defined by $THI=T-0.55\times$ $(1-RH/100)\times (T-14.4)$, where *T* denotes temperature in °C and *RH* denotes relative humidity in %; *WCI* is defined as $WCI=-(10\sqrt{V}+10.45-V)\times (33-T)+8.55S$, where *V* is the wind velocity in m/s and *S* is the sunshine duration in hour per day. The mild weather frequency in percentage in a particular season (year) is the proportion of mild days to all observed days in that season (year).

### Statistical analysis

The long-term trend of mild weather is estimated by the Theil–Sen estimator (also known as Sen’s slope)^30^, and its significance is tested by the modified nonparametric Mann-Kendall trend test^31^. The spatial distributions of the climatology and trends are plotted by interpolating stations to regular grids using the natural neighbor interpolation method^32^. National mean is obtained by averaging all 5-by-5 degree grids with at least three stations. For each grid, its mild weather is obtained by averaging all stations within that particular grid. National population exposure in person-days is simply estimated by multiplying population in China by the national mean frequency of mild weather days^16,33^. All figures were created using MATLAB R2017a.

Regionalization of mild weather is based on *k*-means clustering^34^ of the monthly mild weather frequency of all stations. The “best” number of clusters (*k*) is selected by examining the changes of the sum of squared errores^35^ with increasing *k* from 2 to 15, and the *k*-value at a significant elbow is considered as the “best” *k* (see Fig. 2a).

To assess the possible effects of individual variables (i.e., temperature, relative humidity, wind speed, and sunshine duration) on the long-term trend of mild weather frequency, we recompute the mild weather frequencies under four additional scenarios using a method similar to the ‘factor separation method^16,17^’. In each computation, we allow one variable to evolve across the time but keep others at the climatological levels (i.e., multi-year mean over the base period of 1971–2010). Then the trend from this new computation can be considered as the contribution of the corresponding variable to the trend of mild weather^16,17^.

## Supplementary information

Supporting Information
