# Supplementary material for: Mild weather changes over China during 1971–2014: Climatology, trends, and interannual variability
Source: Sci Rep. 2019 Feb 20;9:2419. doi: 10.1038/s41598-019-38845-8 (PMC6382875; doi:10.1038/s41598-019-38845-8)
Supplement: Supplementary file 1 — Supporting Information [file 41598_2019_38845_MOESM1_ESM.docx]

Supporting Information

**Mild weather changes over China during 1971−2014**

Lijie Lin^1^, Erjia Ge^2^, Chongcheng Chen^3^, Ming Luo^3,4,5*^

^1^School of Management, Guangdong University of Technology, Guangzhou, 510520, China

^2^Dalla Lana School of Public Health, University of Toronto, Toronto, ON, Canada.

^3^Key Laboratory of Spatial Data Mining & Information Sharing of Ministry of Education, National Engineering Research Centre of Geospatial Information Technology, Spatial Information Research Centre of Fujian Province, Fuzhou University, Fuzhou 350116, Fujian, China.

^4^School of Geography and Planning, Sun Yat-Sen University, Guangzhou 510275, China.

^5^Institute of Environment, Energy and Sustainability, The Chinese University of Hong Kong, Sha Tin, NT, Hong Kong SAR, China.

*Correspondence and requests for materials should be addressed to M. Luo (email: luom38@mail.sysu.edu.cn).

# This file includes the following table and figures (all figures were created using MatLab R2017a):

Table S1. Climate suitability evaluation for human body in China based on the temperature-humidity index (THI) and wind chill index (WCI).

Table S2. Trends of mild weather frequency in percentage (% per decade) in different subregions of China during 1971−1997. Bold indicates significance at the 0.05 level. NC: Northern China, NCP: North China Plain, SC: South China, SWC: Southwest China, TP: Tibetan Plateau, YRB: Yangtze River Basin.

Table S3. Same as Table S2 but for 1998−2014.

Figure S1. Time series of annual mean mild weather frequency in percentage in different subregions of China from 1971 to 2014. The black straight line indicates the corresponding trend in 1971–2014, blue denotes the trend in 1971–1997, and red denotes the trend in 1998–2014. Shading represents the 95% confidence interval.

Figure S2. Same as Figure S1 but for spring.

Figure S3. Same as Figure S1 but for summer.

Figure S4. Same as Figure S1 but for autumn.

Figure S5. Same as Figure S1 but for winter.

Table S1. Climate suitability evaluation for human body in China based on the temperature-humidity index (THI) and wind chill index (WCI).

| Level | Suitability | THI | WCI |
| --- | --- | --- | --- |
| 1 | Chilly | < 14.0 | < –400 |
| 2 | Cold | 14.0 ~ 16.9 | –400 ~ –300 |
| 3 | Mild | 17.0 ~ 25.4 | –299 ~ –100 |
| 4 | Hot | 25.5 ~ 27.4 | –99 ~ –10 |
| 5 | Sultry | > 27.5 | > –10 |

Table S2. Trends of mild weather frequency in percentage (% per decade) in different subregions of China during 1971−1997. Bold indicates significance at the 0.05 level. NC: Northern China, NCP: North China Plain, SC: South China, SWC: Southwest China, TP: Tibetan Plateau, YRB: Yangtze River Basin.

|  | China | SWC | TP | NC | NCP | YRB | SC |
| --- | --- | --- | --- | --- | --- | --- | --- |
| Annual | **1.01** | **1.44** | 0.28 | **1.22** | **1.10** | 1.34 | 0.67 |
| Spring | 0.72 | 0.71 | 0.10 | **1.03** | **1.86** | 0.97 | -1.29 |
| Summer | **1.82** | 0.39 | 0.84 | **3.06** | 1.20 | 0.90 | -2.46 |
| Autumn | **1.08** | 1.82 | 0.17 | 0.71 | 1.16 | 2.97 | **3.50** |
| Winter | **0.42** | **2.85** | 0.03 | **0.06** | **0.17** | 0.53 | 2.93 |

Table S3. Trends of mild weather frequency in percentage (% per decade) in different subregions of China during 1998−2014. Bold indicates significance at the 0.05 level.

|  | China | SWC | TP | NC | NCP | YRB | SC |
| --- | --- | --- | --- | --- | --- | --- | --- |
| Annual | **-0.58** | **-4.44** | 0.22 | 0.04 | -0.14 | -2.03 | **-4.15** |
| Spring | -0.06 | -1.76 | -0.07 | 0.45 | 0.95 | -0.55 | -1.40 |
| Summer | **1.39** | -0.31 | 2.13 | 2.72 | 2.28 | -1.49 | 0.09 |
| Autumn | -1.37 | **-4.54** | **-0.77** | -2.34 | -1.69 | -1.66 | -2.03 |
| Winter | **-2.28** | **-11.15** | **-0.40** | **-0.65** | **-2.12** | **-4.41** | **-13.29** |


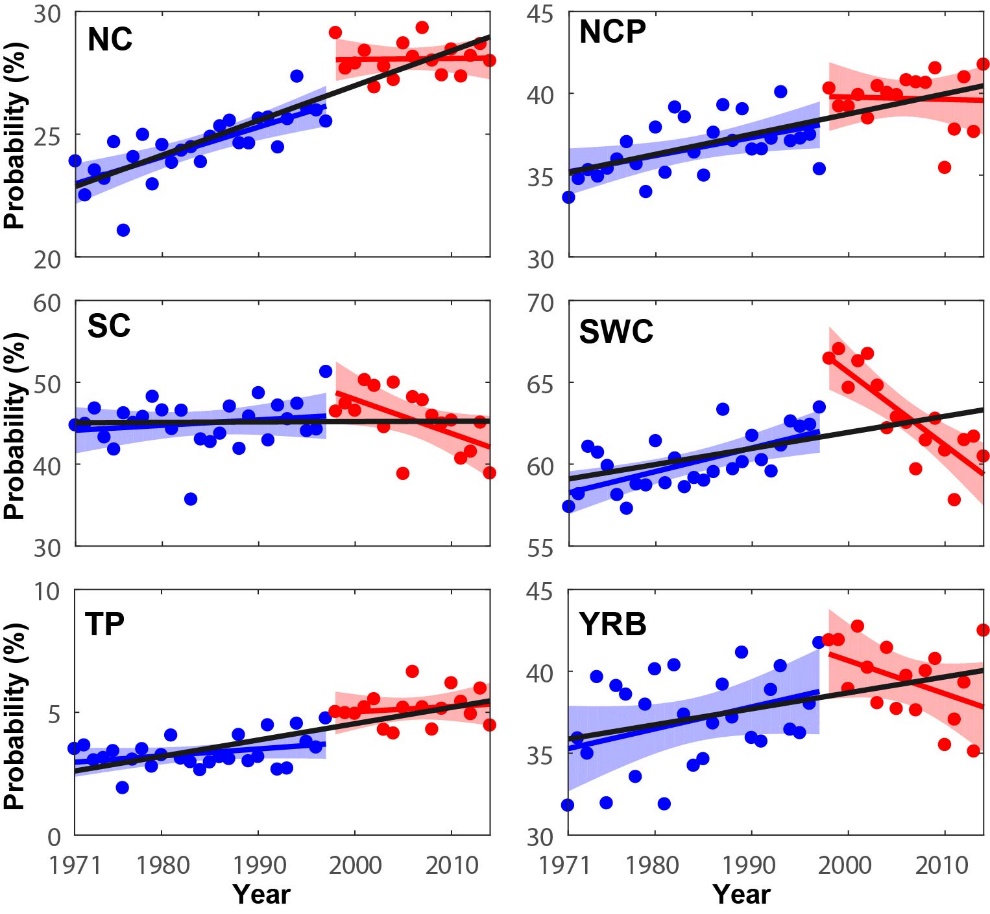


Figure S1. Time series of annual mean mild weather frequency in percentage in different subregions of China from 1971 to 2014. The black straight line indicates the corresponding trend in 1971–2014, blue denotes the trend in 1971–1997, and red denotes the trend in 1998–2014. Shading represents the 95% confidence interval.


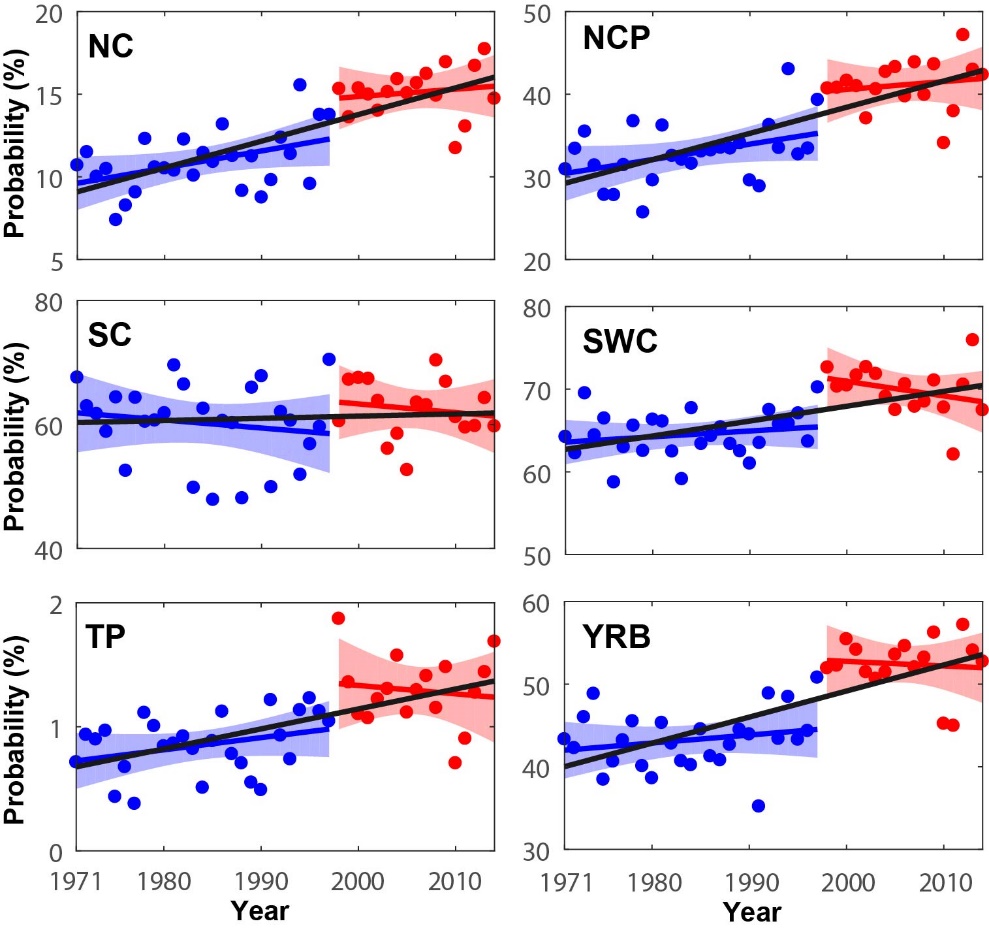


Figure S2. Same as Figure S1 but for spring.


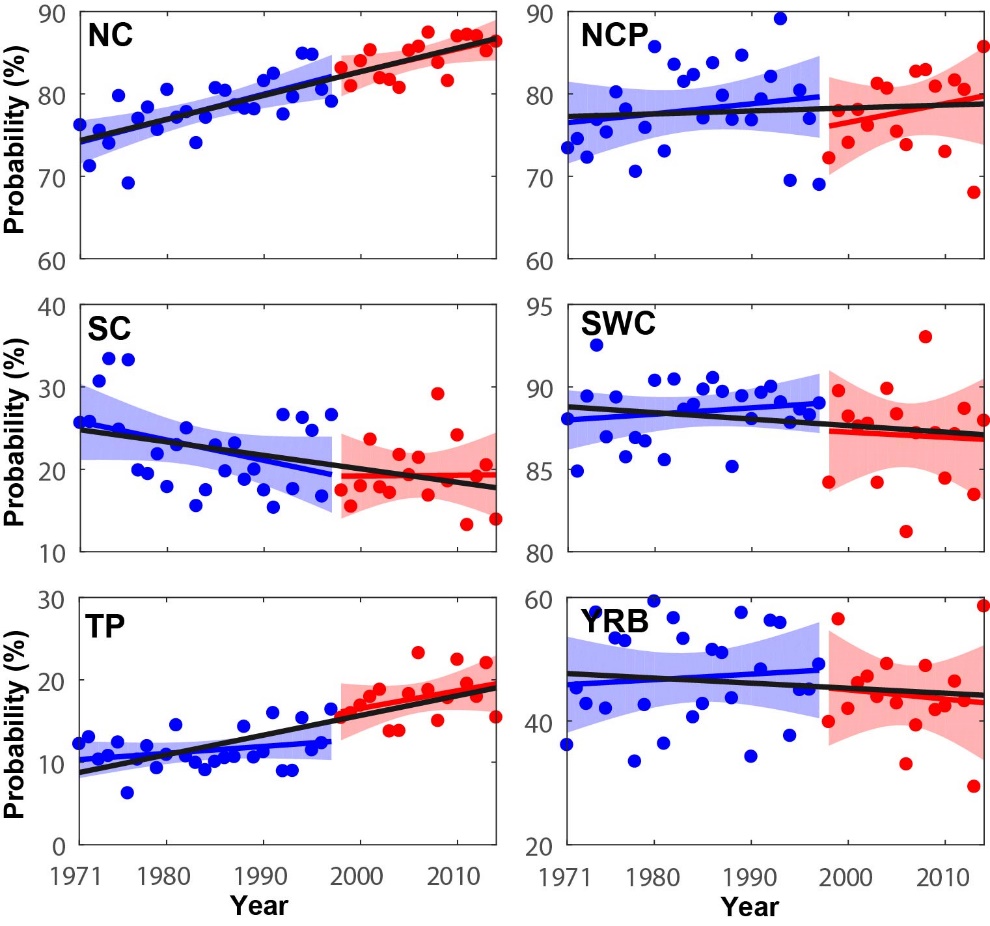


Figure S3. Same as Figure S1 but for summer.


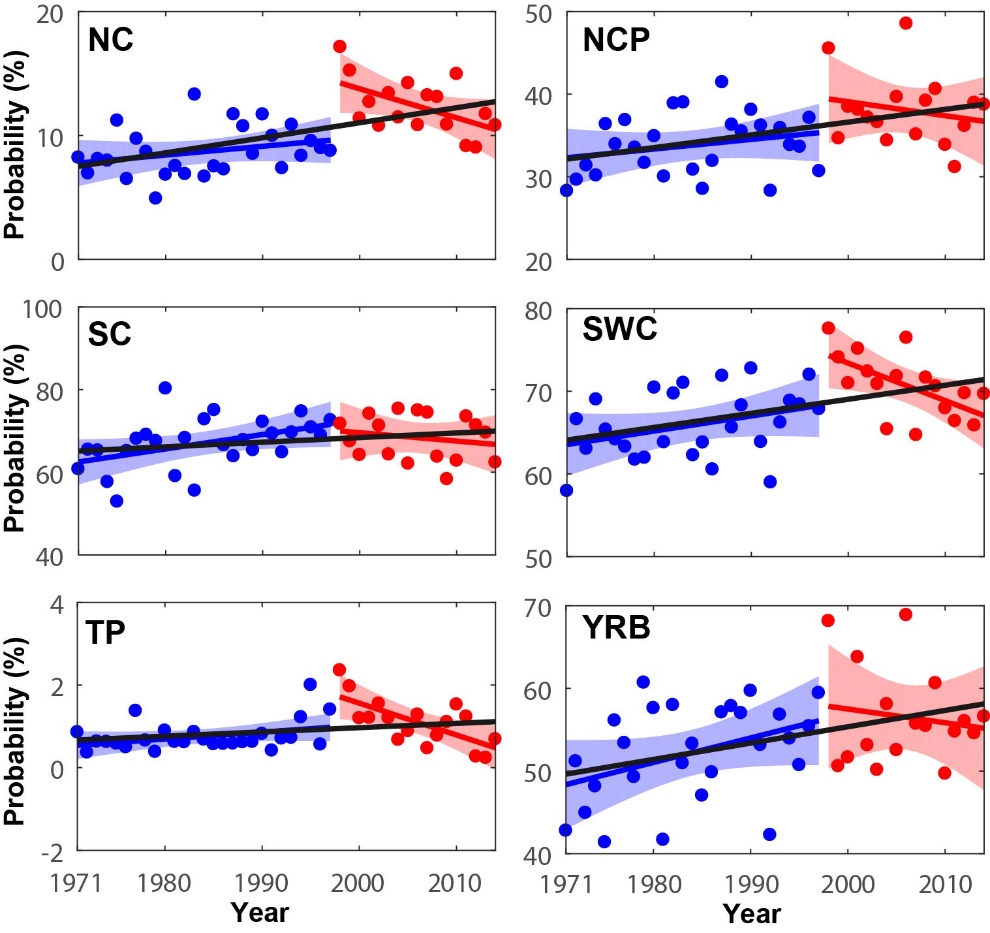


Figure S4. Same as Figure S1 but for autumn.


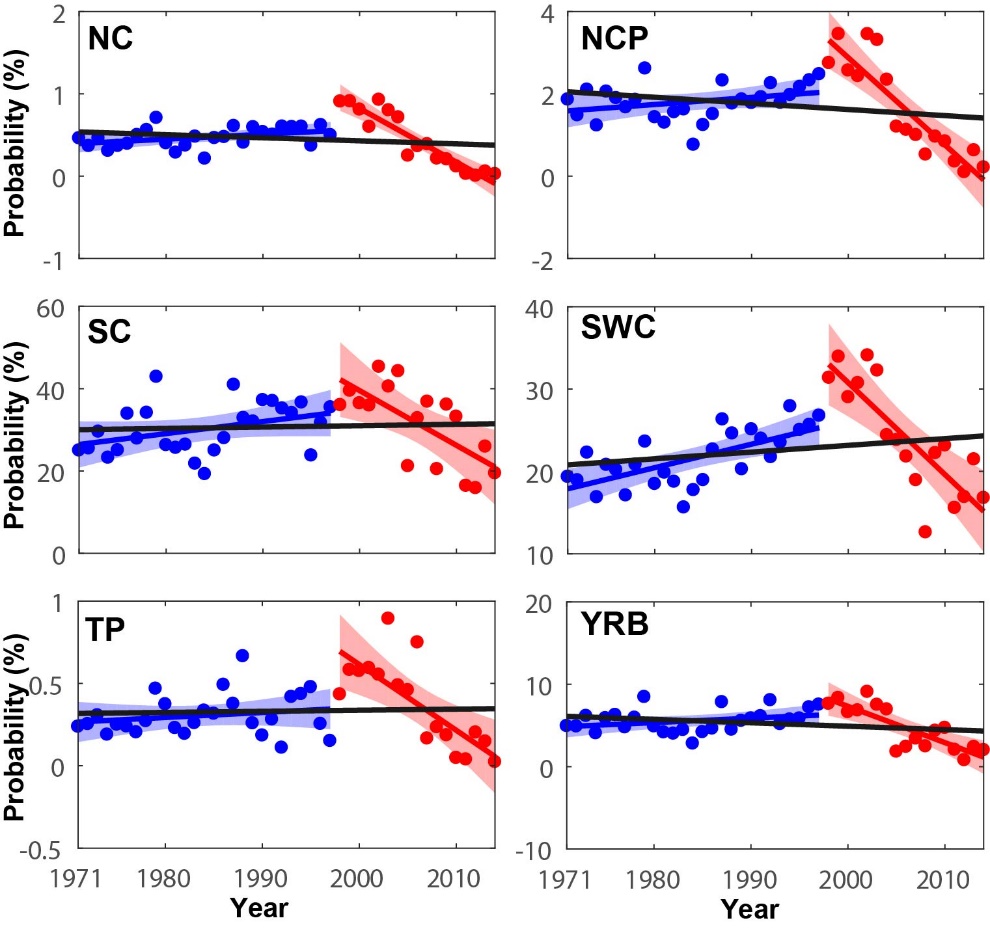


Figure S5. Same as Figure S1 but for winter.
